# Hospitality’s ethical values and unethical employee behaviour: The mediating roles of work values and the moderating role of perceived organisational support

**DOI:** 10.3389/fpsyg.2022.1063797

**Published:** 2022-11-28

**Authors:** Qiuping Chen, Zijuan Liu

**Affiliations:** College of Tourism, Huaqiao University, Quanzhou, Fujian, China

**Keywords:** Hospitality’s ethical value, unethical behaviour, work values, perceived organisational support, ethical decision, hotel industry

## Abstract

In recent years, hotels have occasionally engaged in unethical behaviour. This has become an urgent problem that requires a solution. Based on social exchange theory, this study constructs a theoretical model of the relationship between hospitality’s ethical values and unethical behaviour. According to 543 questionnaires, the findings indicate that hospitality’s ethical values negatively affect the unethical behaviour of employees. Work values played a part in the intermediary role between the two, and perceived organisational support significantly positively moderated the relationship between hospitality’s ethical values and unethical behaviour. By exploring the logical relationship between hotels’ and employees’ morality, this study expands the research content and theoretical framework of unethical employee behaviour and helps to bridge the work values of hotels and individuals. Furthermore, it helps to build a good hotel ethical value system, which can effectively reduce and suppress the emergence of unethical employee behaviour.

## Introduction

Given the increasing complexity of business ethics in organisations and frequent unethical employee practices, effectively reducing unethical employee behaviour has become an important issue for management (Dimitriou and Ducette, 2018; Martínez et al., 2021). In 2017, many well-known five-star hotels, such as Kempinski, Shangri-La and Sheraton, experienced sanitary incidents; in 2019, 25 employees of the OYO Hotel at China subsidiary were fired for “unethical behaviour.” It is not difficult to find that the emergence of various unethical behaviours of hotel employees exposed the serious lack of ethics in the hotel industry; thus, management should change its attitude towards ethical issues. Due to the lack of ethical norms, the hotel industry does not pay enough attention to moral issues, and there are still many moral dilemmas (Garba et al., 2018), which not only affect employee performance (Fehr et al., 2019) but are also detrimental to hotel financial growth and sustainability (Chun et al., 2013). Therefore, the results shown in this study could serve as an empirical basis for guiding hotels and employees to cope with unethical behaviour from having a “deaf ear” to “active inhibition.”

The hotel industry is labour intensive with a complex staff composition that involves not only cross-cultural problems but also the mobility and diversification of customers, which are distinctive characteristics of the hotel industry (Cheng et al., 2013; Schwepker Jr and Dimitriou, 2021). Due to the close contact between staff members and customers, more temptations and weaknesses in management will arise when facing complicated and heavy workloads, resulting in frequent unethical employee behaviours (Cheng et al., 2013; Schwepker Jr and Dimitriou, 2021). Therefore, it is urgent to clarify the avoidance strategies and logics of the unethical behaviour of hotel employees. The premise of effectively reducing and suppressing unethical employee behaviour is to clarify its inducing factors and generation process. The organisational situation is the main factor affecting employee behaviour (Anderson and Burchell, 2019). Corporate ethical values (CEV), as a part of organisational culture, is an important factor affecting the development of employees’ moral habits and is composed of formal and informal ethical policies of enterprises and the moral values of enterprise managers (Hunt et al., 1989). Although previous studies have pointed out that higher corporate ethical values may inhibit employees from engaging in behaviours that are not socially ethical or that do not comply with ethical standards (Tang et al., 2016), it is only in the finance or procurement industry (Baker et al., 2006; Tang et al., 2016) rather than targeting the hotel industry (Dimitriou and Ducette, 2018). Therefore, in the hotel industry, both the relationship and the formation mechanism between hospitality’s ethical values and employee unethical behaviour are unclear (Chen and King, 2018). There is a lack of needed empirical research.

Unethical behaviour does not occur gratuitously. Ethical decision-making theory holds that ethical behaviour is the result of ethical decision-making and a series of psychological processes produced by individuals when facing ethical dilemmas (Schwartz, 2016). The stimulation of internal traits, induction of external factors and ethical events themselves are prerequisites to unethical behaviour. According to the literature, there are three main factors affecting unethical employee behaviour in an organisation: “bad apples” that emphasise individual characteristics and differences (Small and Lew, 2019; Bell and Showers, 2020), “bad situations” that emphasise specific situations and events themselves (Zhang N. et al., 2019; Markiewicz and Czupryna, 2020) and “bad barriers” that emphasise the influence of external factors such as the organisation (Cialdini et al., 2019; Schwepker et al., 2020). It can be seen that the unethical behaviour of employees is the result of a combination of various factors. Both individual characteristics and organisational context are important factors affecting unethical employee behaviour (Anderson and Burchell, 2019). Previous scholars mostly conducted unethical behaviour studies from a single level, such as individuals or organisations, without a comprehensive exploration. In addition, corporate ethical values are a part of corporate culture, which helps to shape employee work values and influence employee behaviour (Jaw et al., 2007). Organisational support is regarded by employees as an incentive and has an important impact on employee positive behaviour (Hur et al., 2013). Thus, this study aims to offer more details to better explore the influence mechanism of both levels on unethical employee behaviour; the influence of hospitality’s ethical values, perceived organisational support and personal work values on unethical employee behaviour are also discussed, to understand the conduction role of organisational factors on individual factors in the influence of unethical behaviour.

Furthermore, based on the social exchange theory, this study tries to build a theoretical model between hospitality’s ethical values and unethical employee behaviour and explore prevention mechanisms and strategies for hotel employees’ unethical behaviour by verifying the mediating role of work values and perceived organisational support. Theoretically, it analyses the role of hospitality’s ethical values, work values and perceived organisational support in the prevention of unethical behaviour among hotel employees, extends the study of the conduction mechanism between hospitality’s ethical values and unethical behaviour among employees, enriches the moral exploration in the hotel field and provides a reference for managers on how to build a good ethical value system for hotels to prevent unethical behaviour among employees (Stevens, 1997).

## Theoretical background and hypothesis development

### Social exchange theory

Social exchange theory was developed in the 1950s in the field of social psychology and has had a profound impact on social interaction research. The rationale of social exchange theory is that interactions between people are all based on the principle of reciprocity and that social behaviour is the result of the exchange process (Akarsu et al., 2020). When employees perceive resources or rewards from the organisation, they give certain rewards in their work, such as work performance (Jung et al., 2021) and extrarole behaviour (Garba et al., 2018). Therefore, social exchange theory provides appropriate theoretical support for exploring the impact of organisational factors on unethical employee behaviour. First, social exchange theory holds that employee attitudes and behaviour are the result of an exchange between employees and organisations. Therefore, unethical employee behaviour is understood as affected by the social exchange within the unethical hotel context (Haldorai et al., 2020). When hotels emphasise ethics in their exchange with employees, employees will suppress their unethical behaviour (Lin et al., 2016). Second, work values, as the evaluation of the working environment, are not only deeply affected by the corporate climate but are also decisive factors that affect employees’ cognitive processes (Saito et al., 2021). The indirect transmission process cannot be separated from the result of the exchange. Finally, perceived organisational support is the subjective perception of the resources that the organisation provides. When employees perceive support from the organisation, they may reduce their own unethical behaviour in return (Côté et al., 2020). Hence, it is argued that employees will be more satisfied with the working environment when enterprises create an ethical climate. Likewise, unethical employee behaviour may be inhibited in a fair working environment.

### Research hypothesis

#### Employee unethical behaviour

Unethical behaviour (UB) in the workplace has recently become increasingly prominent, especially in the hotel industry. It has become a real problem for modern organisations to solve. Employee unethical behaviour is defined as behaviour carried out by employees that violates widely recognised ethical standards and business ethics, such as cheating and lying (Treviño et al., 2006). Unethical employee behaviour, such as theft, neglect and concealment, is common in hotels (Ghosh and Shum, 2019). Such behaviours not only make organisation members engage in more self-interested negative behaviours but also affect the long-term performance and sustainability of the organisation. This problem has attracted widespread attention in academia and industry (Kish-Gephart et al., 2010).

Given the negative effects of employee unethical behaviour, academia has conducted extensive theoretical analyses of its inducing factors and influencing effects to clarify the antecedents of unethical behaviour, including individual characteristics, organisational context and leadership factors, such as five-factor model personality traits (Helle et al., 2018), Machiavellian personality (Zagenczyk et al., 2014), ethical climate (Haldorai et al., 2020), leadership-member exchange (Schwepker and Good, 2017) and moral leadership (Ahmad, 2018). The consequences of unethical behaviour mainly focus on social infectivity (Wiltermuth et al., 2017), shame and guilt (Umphress and Bingham, 2011) and organisational performance (Berry et al., 2007).

A literature review found the following gaps in previous studies. (1) The existing studies on unethical employee behaviour are less related to the service reception industry, especially in the hospitality field. (2) Studies of unethical behaviour discuss the level of individual characteristics and organisational situations separately, and it is less common to combine the two factors to study the effect of organisational factors on individual factors. (3) Although many studies have explored unethical employee behaviour based on the perspective of social exchange, hardly any studies consider the possible impact of two relevant variables, perceived organisational support and work values. To address these problems, our study constructs a model framework for research on unethical behaviour among hotel employees.

#### Hospitality’s ethical values and employee unethical behaviour

Corporate ethical value (CEV) is composed of formal and informal ethical policies of enterprises and the moral values of managers (Hunt et al., 1989). Corporate ethical value is an important factor in determining ethical judgement, which will improve employee performance (Sharma, 2016) and promote the sustainable development of organisations (Lee, 2020). The research conducted by Van Gils et al. (2015) in the United Kingdom and the United States (2015) showed that the ethical organisational climate plays an important role in ensuring ethical decision-making. Specific ethical programs (e.g. ethical norms, training about ethics and institutionalised beliefs) can somewhat inhibit ethical rationalisation, change the ethical climate of the organisation and promote the reduction of unethical employee behaviour (Mulder et al., 2020). Moral leadership helps to restrain the unethical behaviour of employees (Wang et al., 2019). Based on this, this study reasoned that as an environmental factor, hospitality’s ethical values are likely to inhibit employee unethical behaviour; thus we assume the following:

*Hypothesis 1:* Hospitality’s ethical values negatively affect unethical employee behaviour.

#### Mediating role of work values

Liang (2012) defined work values (WVs) as evaluation criteria related to the work or work environment for individuals to determine what is “correct” or “the importance of assessing preferences.” In this study, four types of work values proposed by Hofstede (1984) are introduced, including task value (TV), team value (TEV), reward value (RV) and status value (SV), which belong to the incentives of the organisation to achieve greater job satisfaction and less unethical behaviour.

Most current research on values and ethical behaviour focuses on cultural values (Li and Murphy, 2012). However, individual values also play an important role (Arciniega et al., 2019), which differs from individual variability. Personal values are notions that determine individual attitudes, norms, choices and behaviour (Saito et al., 2021). Ralston et al. (2014) demonstrated that differences in individual unethical behaviour were better explained when values were measured from the individual level rather than the cultural level. Additionally, prior studies have shown that employees prefer ethical companies and are more aligned with the organisation when the company owns higher ethical values (Jung et al., 2010). The essence of high work values is that employees are consistent with the enterprise regarding tasks, teams, rewards and status. Tehranineshat et al. (2020) also noted that the ethical atmosphere influenced working values. Therefore, it is believed that the hotel’s ethical values will affect employees’ work values. Thus, the following hypotheses are proposed:

*Hypothesis 2a.* Hospitality’s ethical values positively affect task value.

*Hypothesis 3a.* Hospitality’s ethical values positively affect team value.

*Hypothesis 4a.* Hospitality’s ethical values positively affect reward value.

*Hypothesis 5a.* Hospitality’s ethical values positively affect status value.

From the perspective of social exchange, a certain interest exchange relationship exists between the enterprise and employees, which implies that employees join the enterprise with certain demands and desires, and the enterprise hires employees to survive and develop, inevitably meeting their needs (Li et al., 2021). The organisational climate will affect the formation of individual values, and fairness and equality are the core and foundation of morality (Killen, 2018). This study argues that employees will be more satisfied with the work environment and reduce the unethical behaviour caused by meeting their needs when an enterprise promotes its ethics as a fair organisation. Arciniega et al. (2019) examined the work value as an antecedent of unethical behaviour. However, what makes the difference is that the work value contains four dimensions: openness to change, conservation, self-enhancement and self-transcendence, which is similar to task value, team value and status value work values except for reward value in this study. When employees are satisfied with their material compensation, they will be more loyal to their organisation (Guan et al., 2014), and therefore, unethical behaviour will be reduced. Thus, reward value may negatively influence unethical employee behaviour, and the following hypotheses are proposed:

*Hypothesis 2b.* Task value negatively affect employee unethical behaviour.

*Hypothesis 3b.* Team value negatively affect employee unethical behaviour.

*Hypothesis 4b.* Reward value negatively affect employee unethical behaviour.

*Hypothesis 5b.* Status value negatively affect employee unethical behaviour.

Task value refers to employees’ work autonomy, challenges and potential realisation. It can be found that the higher the corporate ethical values are, the more attention the enterprise pays to people orientation; the more resources the organisation provides for employees, the greater the work autonomy and the more employees’ self-potential will arise (Dhar, 2016). Previous studies have indicated that a high level of job autonomy can increase creativity and satisfaction; in return, employees may consciously reduce unethical behaviour (Deci et al., 2017). In addition, people who attach importance to self-improvement tend to pursue achievements by manipulating people and resources, making them more likely to engage in unethical behaviour (Saito et al., 2021). However, if hotels provide a fair and ethical environment, unethical behaviour will be reduced accordingly. Therefore, the following hypothesis is proposed:

*Hypothesis 2.* Task value mediates the relationship between hospitality’s ethical values and employee unethical behaviour.

Team value alludes to whether individuals can live and communicate with colleagues harmoniously and honestly. The ethical climate impacts trust in colleagues (Nedkovski et al., 2017), and trust is the foundation of teamwork (Jones and George, 1998); therefore, corporate values may be one of the major indicators to measure team value. Given the nature and demands of the hotel, internal service quality is crucial to the hotel staff, who needs strong support from colleagues and leaders when facing high work requirements and pressure (Wu et al., 2021). Past studies have realised that workplace friendships reduce deviant behaviour (Zhuang et al., 2020) when employees can work together with colleagues. It not only means that the organisation has a good team atmosphere but also improves work performance, making employees more satisfied. Thus, employees will reduce their unethical behaviour. The following hypothesis is proposed:

*Hypothesis 3.* Team value mediates the relationship between hospitality’s ethical values and employee unethical behaviour.

Reward value denotes satisfaction with material rewards. Employees’ satisfaction will increase when they receive reasonable rewards, and the corporation is ethical and fair (Brown and Treviño, 2006). Generally, compensation is a symbol of achievement, representing an organisation’s recognition of employees. Improving wages and benefits is an important means to alleviate hotel employee turnover (Guan et al., 2014). In fact, remuneration in the hotel industry is not considerable, and employees’ job satisfaction will be decreased for unreasonable or low income (Díaz-Carrión et al., 2020). Therefore, providing that hard work is not rewarded and recognised, it is probable for employees to engage in unethical behaviour (Leana and Meuris, 2015) due to their self-interest. However, if an enterprise offers employees fair and reasonable salaries and rewards, employees will naturally reduce their unethical behaviour for selfish gain. Therefore, the following hypothesis is proposed:

*Hypothesis 4.* Reward value mediates the relationship between hospitality’s ethical values and employee unethical behaviour.

Status value implies an assessment of employee satisfaction with promotion opportunities and fairness. In the hotel industry, promotion is not always considered fair, which means that building a fair promotion mechanism can advance hotel employees’ work participation and productivity (Russen et al., 2021). Ethical corporations advocate a fair organisational atmosphere (George Jr, 2021) to make employees believe their organisation has provided fairness in promotion opportunities. Likewise, procedural fairness boosts employees’ motivation to support an organisation (van Dijke et al., 2012). Promotion focus is associated with unethical pro-organisational behaviour (Graham et al., 2015). Individuals who focus on promotion are better at observing and discovering status-related opportunities and are more likely to engage in unethical behaviour for selfish reasons. When hotels supply employees with fair opportunities for promotion, employees are more satisfied and reduce their unethical behaviour out of long-term consideration of the organisation. Thus, the following hypothesis is proposed:

*Hypothesis 5.* Status value mediates the relationship between hospitality’s ethical values and employee unethical behaviour.

#### Moderating role of perceived organisational support

In 1986, Einsberger et al. clarified the concept of perceived organisational support (POS), which refers to the comprehensive perception that an employee develops in the course of his or her work of how much the organisation values his or her contribution and well-being. Hur et al. (2013) found that perceived organisational support plays an important role in determining employee attitudes and behaviours. As a kind of work resource, organisational support can reduce the negative impact caused by resource depletion, such as burnout and unethical behaviour (Chiu et al., 2015). Thus, understanding the psychological processes behind perceived organisational support has a positive impact on both employees (e.g. increased satisfaction, reduced unethical behaviour) and organisations (e.g. improved financial performance; Cheng and Yi, 2018; Chen and Eyoun, 2021).

Studies have shown that perceived organisational support is important in adjusting organisational relationships and affects employee working attitudes and behaviour (Palmer et al., 2017). However, no study has explored the moderating role of organisational support between corporate ethical values and the unethical behaviour of employees. According to social exchange theory, employee attitudes and behaviour depend on the treatment of the organisation (Duan et al., 2021). When employees perceive the organisation’s support, they will also become more proactive, following the principle of reciprocity and reducing their negative behaviour towards the organisation (Chiang and Hsieh, 2012). Therefore, our study predicts that perceived organisational support promotes the impact of hospitality’s ethical values on unethical employee behaviour. In other words, employees will consciously reduce their unethical behaviour in return for organisational support resources. Therefore, the following hypothesis is proposed:

*Hypothesis 6.* Perceived organisational support will negatively moderate the relationship between hospitality’s ethical values and unethical employee behaviour.

## Methodology

### Sample and data collection

To ensure the quality of the collected questionnaires, the research purpose and survey process were explained to each respondent before the survey, emphasising the anonymity of the questionnaire. Since the original scales were in English, the wording was modified in the translation process to adapt to the Chinese situation and then was fine-tuned according to the results. With the help of the hotel human resources manager, questionnaires were collected in the form of anonymous collection boxes to avoid the influence of social praise. The formal survey data were collected from October to November 2020. The research sample includes high-star hotel employees in Fujian, Shanxi, Tianjin, Beijing and Guangdong, among other places. Questionnaires were collected online and offline. A total of 600 questionnaires were distributed, and 557 were returned (92.83%). Finally, invalid questionnaires with obvious regular answers or incomplete answers were eliminated, and 543 questionnaires with an available response rate of 97.49% were coded and analysed in this study.

The demographic characteristics are consistent with the staffing situation of high-star hotels, and the results are shown in Table 1.

**Table 1:** Background of participants (*N* = 543).

Variable	Items	Quantity (%)	Variable	Items	Quantity (%)
Gender	Male	252 (46.4%)			
	Female	291 (53.6%)	Tenure	Below 1 year	164(30.2%)
				1–5 years	157(28.9%)
Age	18–25	216 (39.8%)		6–10 years	151(27.8%)
	26–30	176 is (32.4%)		11–15 years	56(10.3%)
	31–40	106 (19.5%)		16–20 years	12(2.2%)
	41–50	34 (6.3%)		More than 20 years	3(0.6%)
	51–60	11 (2.0%)			
			Rank	Internship	123(22.7%)
Education Background	Junior high school or below	75 (13.8%)		Labour Staff	270(49.7%)
	Senior high school	136 (25.0%)		Junior Managers	90(16.6%)
	College	205 (37.8%)		Middle Managers	45(8.3%)
	University	114 (21.0%)		Senior Managers	15(2.8%)
	Master or Doctor	13 (2.4%)			

### Measurements

The study was conducted on a seven-point Likert scale [ranging from “strongly disagree”(1) to “strongly agree”(7)]. All measurement items used were from previous studies, and the wording was modified to suit the research environment. The questionnaire consists of two parts. The first part contains all the questions about hospitality’s ethical values, the four dimensions of work values, perceived organisational support and unethical behaviour. The second part involves demographic characteristics as the control variable of this study. Details are as follows:

To measure hospitality’s ethical values, this study adapted the scale of corporate ethical value (CEV) compiled by Hunt et al. (1989), consisting of three questions. Relevant studies have also verified its reliability (Sharma, 2016). In this study, hospitality’s ethical values include the organisation’s perception of ethical issues and the extent to which it acts in an ethical manner. A typical item is “Top management has been very clear that unethical behaviour will not be tolerated.”

The four dimensions of work values were measured according to the survey of Hofstede (1984). None of the components of the scale (task value, team value, reward value and status value) show a difference by gender (Cheung and Scherling, 1999) and are suitable for measuring the work value of hotel employees (Cheng et al., 2013). Task value was measured by asking respondents to evaluate aspects of autonomy, work challenges and potential realisation. Team value was measured in terms of cooperation with colleagues, such as harmonious coexistence and candid communication with coworkers. Reward value was evaluated from the perspective of reasonable salary and high salary, while status value was evaluated from the perspective of opportunity fairness and promotion opportunity.

The assessment of perceived organisational support was adapted from the scale of Eisenberger et al. (2001), consisting of five items. Cheng and Yi (2018) verified its reliability in the study of hotel employees’ work results, with the representative item: “The company truly cares about my well-being.”

The scale for measuring employee unethical behaviour was derived from the scale of Dove (1988), which contains 17 items covering a variety of business practices that constitute ethical problems. This is a scale used to measure unethical behaviour in organisations and has been widely used in subsequent studies (Kuenzi et al., 2020). To reduce the impact of social approval, unethical behaviour was measured by reverse questioning. Before data analysis, the items were backcoded. A representative item is: “I never use company services for personal use.”

At the same time, this study collected some control variables that may influence unethical behaviour to verify the relationship between variables more accurately, including gender, age, education, working years and rank. The final report includes the data results of the control variables.

### Data analysis

In this study, SPSS 25.0 and AMOS 24.0 statistical software were used for quantitative analysis. First, reliability and validity analyses were used to test the reliability and validity of each project. Second, regression analysis was used to verify the proposed model and hypothesis; that is, descriptive statistics, confirmatory factor analysis and linear regression were used.

The data involved four variables, namely, hospitality’s ethical values, work values, perceived organisational support and unethical behaviour. First, Cronbach’s alpha value was used to measure the reliability of the variables. The results showed that the Cronbach’s alpha values of the four variables ranged from 0.888 to 0.983, indicating that the reliability quality level of the four dimensions was very high, and the research data were true and reliable (Bagozzi and Yi, 1989). Among them, the Cronbach’s alpha values of the four subdimensions of work values are between 0.857 and 0.903, indicating that the reliability of these four subdimensions is also high. Second, the standardised factor loading of each item was obtained by confirmatory factor analysis, and the average variation extraction (AVE) and composite reliability (CR) were calculated. The results are shown in Table 2. The factor loading of each item was higher than 0.6 (Gieling and Ong, 2016), indicating that the topics to which each latent variable belonged were very representative. Meanwhile, AVE and CR also meet the reference standard (Fornell and Larcker, 1981); that is, the convergent validity is good. Finally, the overall fitting coefficient of the four-factor model fits perfectly, so the questionnaire has good construct validity.

**Table 2:** Descriptive statistics and confirmatory factor analysis.

Confirmatory factor analysis (*N* = 543)									
Variables	Items	Factor loading	Cronbach’s *α*		AVE		CR		Overall model fit coefficient
Hospitality’s ethical value	HEV1	0.856	0.888		0.728		0.889		*X*^2^ = 9088.159 *X*^2^/*df* = 1.685 NFI = 0.958 RFI = 0.953 IFI = 0.982 TLI = 0.981 CFI = 0.982 RMSEA = 0.036 PNFI = 0.868 PCFI = 0.890
	HEV2	0.880							
	HEV3	0.823							
Work values	TV1	0.860	0.951	0.907	0.763	0.763	0.970	0.906	
	TV2	0.873							
	TV3	0.888							
	TEV1	0.888		0.907		0.765		0.907	
	TEV2	0.859							
	TEV3	0.877							
	RV1	0.875		0.872		0.774		0.872	
	RV2	0.884							
	SV1	0.870		0.857		0.750		0.857	
	SV2	0.862							
Perceived organisational support	POS1	0.835	0.932		0.734		0.932		
	POS2	0.860							
	POS3	0.870							
	POS4	0.871							
	POS5	0.847							
Unethical behaviour	UB1	0.872	0.983		0.767		0.973		
	UB2	0.847							
	UB3	0.884							
	UB4	0.859							
	UB5	0.850							
	UB6	0.884							
	UB7	0.892							
	UB8	0.882							
	UB9	0.889							
	UB10	0.894							
	UB11	0.878							
	UB12	0.892							
	UB13	0.882							
	UB14	0.891							
	UB15	0.879							
	UB16	0.902							
	UB17	0.884							

Through correlation analysis (Table 3), it can be seen that hospitality’s ethical values, work values, perceived organisational support and unethical behaviour are significantly correlated. The correlation coefficients were all less than the AVE square root, indicating good discriminative validity of the scale.

**Table 3:** Means, standard deviations, correlations and discriminant validity.

Variables	Mean	SD	1	2	3	4	5	6	7
1. Hospitality’s ethical values	4.79	1.71	**0.853**						
2. Task value	4.73	1.74	0.745^***^	**0.873**					
3. Team value	5.01	1.68	0.740^***^	0.785^***^	**0.875**				
4. Reward value	4.57	1.77	0.682^***^	0.734^***^	0.649^***^	**0.880**			
5. Status value	4.74	1.70	0.757^***^	0.771^***^	0.751^***^	0.767^***^	**0.866**		
6. Perceived organisational support	4.53	1.57	0.507^***^	0.514^***^	0.576^***^	0.459^***^	0.538^***^	**0.857**	
7. Unethical behaviour	3.87	1.88	−0.543^***^	−0.576^***^	−0.446^***^	−0.715^***^	−0.604^***^	−0.382^***^	**0.876**

^*^p < 0.05; ^**^p < 0.01; ^***^p < 0.001. The square root of the average variance extraction is shown on the diagonal in bold.

Since the items involved in the study were all self-rated by hotel staff, there may be a problem of homology bias. The Harman single-factor test was first used in this study, and the results showed that the maximum extraction factor could only explain 46.06% (less than 50%) of the variance (Podsakoff and Organ, 1986), so the questionnaire avoided potential homology bias.

## Results

Three analyses were conducted in this study: (1) SEM was used to verify each direct path; (2) a bootstrap confidence interval approach was adopted to test the mediating effect of work values between hospitality’s ethical values and unethical behaviour; and (3) hierarchical regressions in SPSS 25.0 were employed to examine whether perceived organisational support moderates the relationship between hospitality’s ethical values and unethical behaviour.

All path coefficients were significant. Hospitality’s ethical values have a significant negative impact on unethical employee behaviour (*β* = −0.102, *p* < 0.01). Therefore, H1 is supported. There is a significant positive direct impact between hospitality’s ethical values and the four subdimensions of work values (*β*_1_ = 0.745, *p* < 0.001; *β*_2_ = 0.740, *p* < 0.001; *β*_3_ = 0.682, *p* < 0.001; *β*_4_ = 0.757, *p* < 0.001). Thus, H2a, H3a, H4a and H5a are supported. The impact between the four subdimensions of work values and unethical employee behaviour is proved to be significantly negative (*β*_1_ = −0.143, *p* < 0.01; *β*_2_ = −0.225, *p* < 0.001; *β*_3_ = −0.584, *p* < 0.001; *β*_4_ = −0.145, *p* < 0.01), which means Hypothesis2b, 3b, 4b and 5b are supported.

### Mediating effects test

The second analysis verifies whether the four subdimensions of work values mediate the influence of hospitality’s ethical values on unethical behaviour through a bootstrap confidence interval approach. Under 95% confidence, if the confidence interval of bias-corrected and percentile does not contain 0, that is, neither the lower limit nor the upper limit is 0, it can be considered that the mediating effect exists when the confidence is 95% (Hayes, 2017). The results of bootstrap are shown in Table 4. Each confidence interval of bias-corrected and percentile does not contain 0. When four subdimensions are added, the influence between hospitality’s ethical values and unethical behaviour is still significant but significantly weakened. Therefore, the four subdimensions of work values play a partial mediating role between hospitality’s ethical values and unethical behaviour, and the mediating role is RV > SV > TV > TEV; thus, Hypotheses 2–5 are all supported.

**Table 4:** Mediation effect test.

Hypothesis path	Estimates	Bias-corrected 95% CI		Percentile 95% CI		Results
		Lower	Upper	Lower	Upper	
HEV → TV → UB	0.2631	0.1807	0.3543	0.1803	0.3521	Supported
HEV → TEV → UB	0.0848	0.0075	0.1717	0.0058	0.1653	Supported
HEV → RV → UB	0.3817	0.3102	0.4540	0.3093	0.4582	Supported
HEV → SV → UB	0.3053	0.2192	0.3928	0.2197	0.3933	Supported

### Moderating effects test

**Figure 1:**
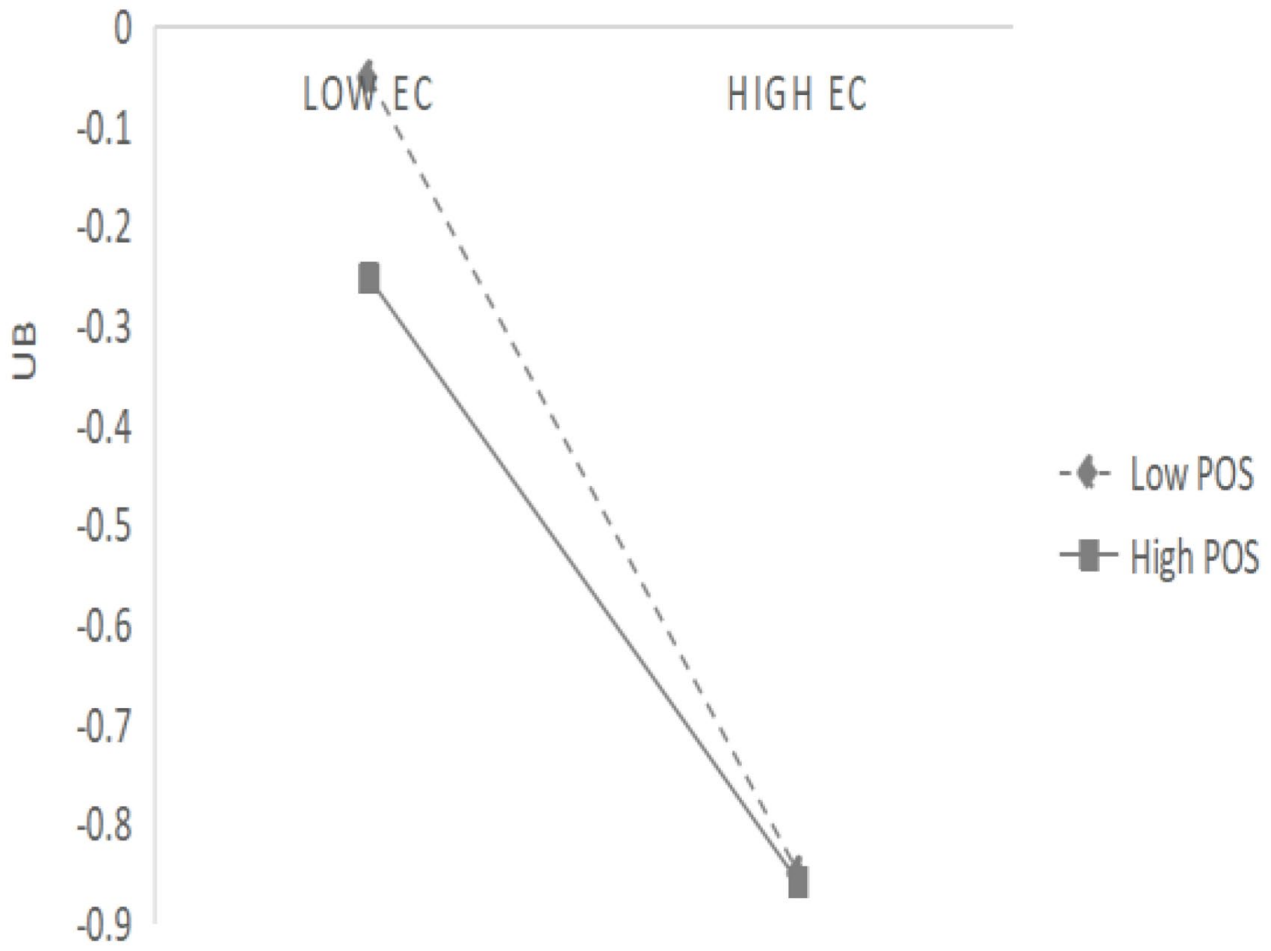
Moderating effect diagram (POS).

The third analysis verified the moderating effect of perceived organisational support on the impact of hospitality’s ethical values on unethical behaviour by hierarchical regression. The results are shown in Table 5. When perceived organisational support was used as a moderating factor, the *F* value changed significantly (*F* = 43.143^***^), and its interaction item (HEV*POS) was also significant (*β* = 0.096, *t* = 2.733^**^), indicating that the positive interaction effect of HEV*POS on unethical behaviour is significant. Therefore, H6 is supported. To explain this effect more intuitively, this study used a simple slope graph to describe the changes in the relationship between hospitality’s ethical value and unethical behaviour at different levels of organisational support (Figure 1). According to the slope diagram, the greater the perceived organisational support, the more obvious the negative influence relationship between hospitality’s ethical values and unethical behaviour. In other words, when hotels have the same level of ethical value, the lower the perceived organisational support, the more unethical the behaviour are.

**Table 5:** Results of the moderator.

Variables	M1	M2	M3
Gender	0.088	0.092	0.094
Age	−0.147^***^	−0.143^***^	−0.137^***^
Education	0.072^*^	0.079^*^	0.077^*^
Tenure	−0.124^*^	−0.113^**^	−0.122^***^
Rank	−0.069	−0.074	−0.078^*^
HEV	−0.497^***^	−0.436^***^	−0.399^***^
POS		−0.126^**^	−0.105^**^
HEV*POS			0.096^**^
*R* ^2^_adj_	0.366	0.376	0.383
*F*	53.047^***^	47.662^***^	43.143^***^

^*^p < 0.05; ^**^p < 0.01; ^***^p < 0.001. HEV is hospitality’s ethical values, POS is perceived organisational support.

In summary, hospitality’s ethical values are a significant negative predictor of unethical behaviour, and work values play a partial intermediary role between the two, while perceived organisational support negatively moderates the relationship between hospitality’s ethical values and unethical behaviour.

### Robustness checks

To test the robustness of the research model, the same regression analysis and SEM method were used for verification. Work values are evaluated in another way; that is, their four subdimensions are combined into a factor for model replacement (Zhang S.-N. et al., 2019). The results showed that all the paths of the alternative model were significant, but the overall fitting effect was lower than that of the original model (*X*^2^ = 1509.033, *p* < 0.000, *X*^2^/*df* = 2.724, NFI = 0.930, RFI = 0.925, IFI = 0.954, TLI = 0.951, CFI = 0.954, RMSEA = 0.056, PNFI = 0.866, PCFI = 0.889); that is, the original research model has strong robustness.

## Discussion and conclusion

Based on social exchange theory, this study explores the influence process between hospitality’s ethical values and unethical employee behaviour and provides a theoretical reference for the hotel industry to reduce and restrain unethical employee behaviour. Details are as follows:

First, hospitality’s ethical values have a significant negative impact on employees’ unethical behaviour; that is, the greater the hospitality’s ethical values are, the less employees’ unethical behaviour are. This paper further supports the research conclusion of Chen and King (2018) that an effective way to prevent unethical and abnormal behaviour is to establish a clear set of ethical values at the enterprise level. The greater hospitality’s ethical values are, the more standard the hospitality’s ethical policy is and the more moral the managers’ behaviour is. In a moral atmosphere, there is no reason for employees to violate norms and engage in unethical behaviour. Different from Chen and King (2018), this study takes work values as an intermediary variable and proposes a new path for hospitality’s ethical values to influence employees’ unethical behaviour.

Second, in addition to team value, task value, reward value and status value all play a partial mediating role in the relationship between hospitality’s ethical values and employee unethical behaviour. Among them, reward values have the strongest mediating effect, followed by status values and task values. Work values are the criteria for individuals to evaluate their work and work environment and the results they desire to obtain in their work (King et al., 2017). As different means of incentives, material reward is the most direct compensation measure for low-wage hotels. When moral policies are more standardised, the reward and punishment mechanisms are clearer, and unethical behaviour for personal gain is less common. As employees’ perception of the fairness of organisational promotion and whether it meets their potential realisation, status value and task value play a key role in the prediction of employees’ unethical behaviour motivation because fairness and self-realisation are important for personal development (Maxwell et al., 2010). Team values represent the importance individuals attach to whether colleagues get along and cooperate. Establishing a good relationship is conducive to the development of a career (Wang et al., 2020). Therefore, to maintain a good relationship and obey the collective, individuals may engage in some unethical behaviours. Current discussions on work values focus on the Western context, but different from Western individualism, China is a relationship-oriented society that advocates collectivism and “emphasising rites and practising harmony.” Therefore, employees may reduce their egoistic unethical behaviours but may engage in more altruistic unethical behaviours based on reciprocity to gain support from colleagues (Wu et al., 2021). Therefore, the mediating effect of team values was not significant.

Finally, this study further confirms the negative moderating effect of perceived organisational support on hospitality’s ethical values and employee unethical behaviour. Perceived organisational support is the perceived commitment of an organisation to itself. Most employees believe that there is a reciprocal exchange relationship between their work and the organisation, which produces relative dependence outside the formal contract and makes employees feel obliged to respond to the organisation with a positive work attitude and behaviours that are in line with the organisational environment (Cheng and Yi, 2018). Therefore, in hotels with high moral values, the greater the staff perceive support from the organisation, the closer the staff will be to the organisation, inhibiting the unethical behaviours.

### Theoretical implications

First, this study helps to understand and apply ethical issues and work values in the context of Chinese hotels. Unethical behaviours such as cheating, theft and corruption often occur in hotels, but most of the previous studies were conducted in nonhotel environments without considering hotel ethics (Dimitriou and Ducette, 2018). The hotel industry in China lacks guidance of staff’s work values, as well as the emphasis on ethics, punishment and supervision. This study emphasises the importance of the construction of hotel ethics. In addition, studies have shown that there are differences in moral codes among different cultures (Vitolla et al., 2021). Previous studies paid less attention to the work values of employees from different cultural backgrounds (Aladwan et al., 2016). Chinese culture has its uniqueness, emphasising collectivism and paying more attention to the maintenance of work “relationships” while Western countries attach importance to individualism and personal interests. Therefore, the perception of work and needs may be different. Therefore, it is necessary to discuss hotel ethics and employee work values in the Chinese context.

Second, based on social exchange theory, this study constructs a theoretical model of the relationship between hospitality’s ethical values and employees’ unethical behaviour, which enriches the formation mechanism and prevention strategies of employees’ unethical behaviour in the hotel field. As mentioned above, there are few studies on employees’ unethical behaviour that consider the combination of organisational factors and individual factors. This study connects the two by exploring the process by which ethical values at the hotel level affect unethical behaviour through personal work values and verifies the transmission effect of organisational factors on individual factors. Although corporate ethical values and work values are important antecedents of unethical behaviour (Chen and King, 2018; Arciniega et al., 2019), this study found that hospitality’s ethical values have a negative impact on employees’ unethical behaviour through work values. By introducing four kinds of work values to verify the results, the formation process of unethical employee behaviour is clarified. This paper supplements the research on the process path of corporate ethical values and employee unethical behaviour. In addition, as mentioned above, this study also found the particularity in the Chinese cultural context, that is, the mediating effect of team value on the relationship between hospitality’s ethical values and employees’ unethical behaviour was not significant.

Finally, this study further explores the boundary conditions of unethical behaviour by introducing perceived organisational support, which helps to expand the research on the constraint conditions of unethical behaviour. Although extensive studies have explored the moderating effect of perceived organisational support, few studies have been introduced into the study of employee unethical behaviour. This study found that perceived organisational support is also an important moderating factor in the relationship between hospitality’s ethical values and employee unethical behaviour. Perceived organisational support, as a kind of work resource, improves employees’ job satisfaction. Based on social exchange theory and the reciprocity principle, employees will return more positive attitudes and behaviour to the organisation (Li et al., 2019), pay more attention to their long-term interests and reduce their own unethical behaviour. Therefore, this study introduces perceived organisational support to explore the boundary conditions of employee unethical behaviour, which not only expands the positive role of organisational support but also enriches the boundary understanding of employee ethics research.

### Management implications

First, hotels should pay more attention to the construction and practice of corporate ethics. Specifically, hotels should improve their moral training systems and enrich the methods of moral training, such as combining classroom demonstration and role-playing and establishing regular ethics days (Lee et al., 2014). Meanwhile, they should attach importance to the cultivation of moral leaders, the establishment of ethical teams and anonymous moral complaint channels. In addition, the corresponding disciplinary provisions and specific codes of ethics should be further improved. A clear reward and punishment system will help employees and hotels reach a consensus on ethical expectations and create an ethical organisational environment.

Second, hotels should value and guide employees’ work values by developing norms and guidelines. Attention should be given to the influence of development factors, security factors and prestige factors on work values. For example, appropriate delegation of authority can encourage employees to exert their potential and cultivate their ability to deal with difficult problems flexibly and independently to realise task value, which is very important for the new generation of employees (Frye et al., 2020). Low remuneration has always been the main factor affecting the turnover of hotel employees (Patiar and Wang, 2020). Certain material rewards will help employees resist and reduce unethical behaviour, so a reasonable and better salary system and welfare treatment are particularly important. At the same time, hotels should formulate a standardised employment system and a fair promotion mechanism to improve the status value of employees. In addition, hotel human resources should pay attention to the matching of values as selection and recruitment criteria.

Third, hotels should give more support to employees by caring about their well-being and attaching importance to their efforts. For example, hotels can develop appropriate incentives to recognise employees’ contributions to the organisation. Providing employees with resources to help them cope with the pressure of role performance through training, development and coaching is also a good way to improve employees’ well-being (Agarwal, 2020). At the same time, more team-building activities can also be organised to enhance team cohesion and the sense of employee belonging to create a good working environment.

### Limitations and future research

First, the data in this study are limited to high-star hotels in a few regions, without nationwide stratified sampling. In addition, the study is only based on the Chinese cultural context and does not consider the cultures of other countries. Therefore, the representativeness and applicability of samples should be improved in future studies. Second, common method deviation does not exist by examination, but the data are from the same subject, which may lead to homologous deviation. Moreover, as unethical behaviour is a sensitive topic influenced by social praise, future research may consider using a variety of methods, such as the projection method and situational experiments, to improve the validity and objectivity of the measurement results. In addition, this study only verifies the transmission effect of organisational factors (hospitality’s ethical values) on individual factors (work values, unethical behaviour of employees) through SEM. Cross-layer samples can be used for future research to improve the objectivity of the results. Last, among the factors influencing unethical behaviour, there may be other variables that affect the mechanism of these behaviours, such as employees’ emotional orientation, justice sensitivity and responsibility perception. In the future, other multiple paths can be explored to deepen enterprises’ understanding of the boundary conditions of managing such behaviour to better restrain the occurrence of unethical behaviour.

## Data availability statement

The original contributions presented in the study are included in the article/Supplementary material, further inquiries can be directed to the corresponding author.

## Author contributions

QC and ZL conceived the study. ZL collected and analysed the data. QC wrote the manuscript. All authors contributed to the article and approved the submitted version.

## Funding

This paper was supported by the General Project of National Social Science Fund of China (21BGL256).

## Conflict of interest

The authors declare that the research was conducted in the absence of any commercial or financial relationships that could be construed as a potential conflict of interest.

## Publisher’s note

All claims expressed in this article are solely those of the authors and do not necessarily represent those of their affiliated organizations, or those of the publisher, the editors and the reviewers. Any product that may be evaluated in this article, or claim that may be made by its manufacturer, is not guaranteed or endorsed by the publisher.
